# 486. Characteristics Associated with SARS-CoV-2 Infection in Children

**DOI:** 10.1093/ofid/ofab466.685

**Published:** 2021-12-04

**Authors:** F Curtis Sudbury, Amanda Williams, Michelle Kwon, Leah Musser, Patrick Gavigan, Jessica E Ericson

**Affiliations:** 1 Penn State College of Medicine, Hershey, PA; 2 Penn State Children’s Hospital, Hummelstown, PA; 3 Penn State Hershey, Hershey, PA

## Abstract

**Background:**

We sought to describe the range of Acute Respiratory Syndrome Coronavirus 2 (SARS-CoV-2) infection in children.

**Methods:**

Patients < 18 years of age who had a positive nasopharyngeal polymerase chain reaction (PCR) for SARS-CoV-2 at a single health system in central Pennsylvania from 3/19/2020-12/31/2020 were identified. Using a random number generator, 150 additional patients < 18 years of age who had a negative PCR test were also identified. Asymptomatic patients and those without clinical data in the electronic medical record were excluded from analysis. Demographic characteristics, symptoms present at the time of testing, and outcomes were compared between PCR-positive and negative patients. Odds ratios were calculated using univariable and multivariable logistic regression models to patients with positive vs. negative PCR tests.

**Results:**

We included 544 patients in analysis, 412 (76%) of which had a positive SARS-CoV-2 PCR. PCR-positive patients were statistically more likely to have a known contact, no comorbidities, and to present with cough, cold-like symptoms, headache, or loss of taste and smell. All patients who presented with loss of taste and smell were PCR positive at time of presentation. Positive patients were statistically less likely to present with fever or emesis than negative patients. Multivariable regression identified increased age, cough, cold symptoms, headache, and non-white race as predictive of PCR positivity. Patients who tested positive were statistically less likely to be admitted to the hospital and less likely to require respiratory support than negative patients.

**Conclusion:**

Loss of taste and smell is a specific, though uncommon, indicator of SARS-CoV-2 infection in the pediatric population. Headache, cough, and cold-like symptoms are also suggestive of SARS-CoV-2 infection, while fever and gastrointestinal symptoms appear less common. This data suggests that screening questions developed for adults may be less applicable in children. Future research, including more dedicated and prospective studies, is warranted to identify patients in whom a positive SARS-CoV-2 test is sufficiently likely to warrant isolation and testing.

**Disclosures:**

**All Authors**: No reported disclosures

